# Global, regional, and national burden of endometriosis among women of childbearing age from 1990 to 2021: a cross-sectional analysis from the 2021 global burden of disease study

**DOI:** 10.1097/JS9.0000000000002647

**Published:** 2025-06-17

**Authors:** Shuangfei Xu, Yi Zhang, Peng Ye, Qin Huang, Yinan Wang, Yan Zhang, Chaogang Yang, Jinli Ding

**Affiliations:** a Reproductive Medical Center, Renmin Hospital of Wuhan University and Hubei Clinic Research center for Assisted Reproductive Technology and Embryonic Development, Wuhan, China; b Department of Pharmacy, Renmin Hospital of Wuhan University, Wuhan, China; c Department of Gynecology and obstetrics, Renmin Hospital of Wuhan University, Wuhan, China; d Department of Clinical Laboratory, Institute of Translational Medicine, Renmin Hospital of Wuhan University, Wuhan, Hubei, China; e Department of Gastrointestinal Surgery, Zhongnan Hospital of Wuhan University & Hubei Key Laboratory of Tumor Biological Behaviors & Hubei Cancer Clinical Study Center & The Clinical Medical Research Center of Peritoneal Cancer of Wuhan, Wuhan, China

**Keywords:** crosssectional study, disability-adjusted life years, endometriosis, global burden of disease study 2021, incidence, prevalence

## Abstract

**Background:**

Endometriosis is a major contributor to infertility and pelvic pain, which brings a significant burden on family and society. Although the data of endometriosis in Global Burden of Disease (GBD) 2019 was reviewed, no updated analysis has been conducted using GBD 2021, and the disease burden across different age groups has not been analyzed. The aim of this cross-sectional analysis was to provide an up-to-date assessment of the prevalence, incidence, and disability-adjusted life-years (DALYs) of endometriosis from 1990 to 2021 at the global, national, and regional levels.

**Material and methods:**

We obtained data on the prevalence, incidence, and DALYs of endometriosis from GBD 2021. These data were analyzed to provide an overview of the epidemiological trends and disease burden of endometriosis in 204 countries and regions worldwide from 1990 to 2021, and we projected trends through 2040. Health inequality analysis, joinpoint regression analysis, and decomposition analysis were applied to data assessment.

**Results:**

In 2021, the global burden of endometriosis remained substantial, with a total of 22.28 million cases (95% UI: 13.67, 33.69), corresponding to an age-standardized prevalence rate (ASPR) of 1023.8 per 100 000 (95% UI: 627.36, 1549.77). The age-standardized incidence rates (ASIR) was 162.71 (95% UI: 85.21, 265.35) per 100 000, while the age-standardized DALY rate (ASDR) was 94.25 (95% UI: 50.82, 157.73) per 100 000. Regionally, areas with low sociodemographic index (SDI) experienced the highest ASPR, ASIR, and ASDR, while high SDI regions exhibited the lowest rates. Geospatially, Oceania and Eastern Europe displayed the highest ASPR, ASIR, and ASDR. Among countries, Niger had both the highest ASPR and ASDR, and Solomon Islands had the highest ASIR. Women aged 25–29 years emerged as the most affected group, suggesting that this cohort should be a key focus for interventions. By 2040, the global ASPR of endometriosis is projected decline to 887.89 per 100 000 (95% CI: 530.79, 1245), representing a decrease of 13.28% from 2021. Decomposition analysis showed population growth was the major contributing factor, followed by epidemiologic change.

**Conclusion:**

While the burden of endometriosis has decreased globally from 1990 to 2021, significant disparities remain, especially in low SDI regions. It is necessary to develop better policies and preventive measures to effectively address the range of problems associated with endometriosis.

## Introduction

Endometriosis is an estrogen dependent chronic disease, characterized by the presence of endometrial-like tissues outside the uterus. The insidious onset, inadequate disease detection, and lack of noninvasive diagnostic methods lead to delayed diagnosis or even misdiagnosis[1]. The Global Women’s Health Study (GSWH) reported an average diagnostic delay of endometriosis of 3–10 years[2]. The incidence rate of endometriosis has been reported to be increasing in recent years. Data from 2018 indicate that endometriosis affected about 190 million women worldwide, roughly 10 to 15% of women at reproductive age[3]. Notably, since the gold standard for diagnosing of endometriosis is laparoscopic surgery, its true prevalence is unknown[4]. Endometriosis has numerous harmful effects on women. Notably, infertility is a major consequence – approximately 50% of infertile women have endometriosis[5]. In addition, women with endometriosis commonly experience dyspareunia[6], pelvic pain and dysmenorrhea, pregnancy loss, gestational diabetes mellitus and hypertensive disorders of pregnancy[7]. Patients with endometriosis also have a significantly increased risk of depression and anxiety[8], autoimmune diseases[9], migraines[10], breast, or ovarian cancer^[11,12]^. These comorbid conditions can greatly affect personal relationships, educational and employment opportunities, and overall quality of life^[13,14]^. Although endometriosis is a benign disease, its malignant-like behavior – such as invasion, implantation, and recurrence[15] – indicate that prevention of this disease is far more important than treatment.

Endometriosis remains a challenging condition that significantly affects patients’ quality of life and contributes to a substantial long-standing medical burden. The direct medical costs of treating endometriosis, as well as the indirect costs (lost work and productivity), are very high. In fact, the medical costs of endometriosis have been estimated to be similar to those of other chronic conditions such as diabetes and heart disease[16], which ere major threats to the economy and society. The GSWH estimated the average annual cost of endometriosis at $9579, whereas a study from Australian found that the average annual cost of $20 898, with the largest proportion of this cost attributable to lost productivity[2]. Soliman et al. conducted a systematic literature review (2000–2013) of endometriosis costs. They found that the annual direct cost per patient ranged from $1109 (Canada) to $12 118 (USA), and the annual indirect cost ranged from $3314 (Austria) to $15 737 (USA)[17]. These data indicate that the economic burden of endometriosis varies greatly by country but is substantial everywhere. Therefore, comprehensive and up-to-date epidemiological data are indispensable for policymakers and researchers in developing effective response strategies.

Prior to our work, three studies have analyzed the burden of endometriosis. One study focused on the incidence and prevalence rates based on Global Burden of Disease Study (GBD) 2017[18]. Another examined the burden in China and looked only mortality and disability-adjusted life-years (DALYs) rates[19]. The third was a brief global assessment using GBD 2019, reporting on incidence and DALYs[20]. GBD 2021 is a comprehensive global dataset of disease and related injuries[21]. To inform policy with the latest data on endometriosis trends from 1990 to 2021, the present study utilizes the most up-to-date GBD 2021 estimates. This study represents the first comprehensive investigation to utilize decomposition analysis and cross-country inequality assessment in evaluating global trends and disparities in endometriosis burden. Furthermore, we have conducted a pioneering age-stratified analysis of endometriosis burden, offering unprecedented insights into age-specific disease patterns and enabling evidence-based policy formulation for targeted age-group interventions. By utilizing the latest comprehensive data from GBD 2021, the research clarifies the impacts of demographic, age-related, and epidemiological factors on disease burden and provides the first projections of future trends in endometriosis burden.

**HIGHLIGHTS**

- The aim of this study was to provide an up-to-date assessment of the prevalence, incidence, and disability-adjusted life years of endometriosis from 1990 to 2021 at the global, national, and regional levels.
- The burden of endometriosis has decreased globally from 1990 to 2021, mainly due to population growth and epidemiologic change.
- The prevalence rate of endometriosis is projected to decrease by 2040.
- The burden associated with endometriosis primarily affected women aged 25–29 years.

The work has been reported in line with the Strengthening The Reporting Of Cohort Studies in Surgery (STROCSS) criteria[22].

## Materials and methods

### Data sources

All data in this cross-sectional analysis were retrieved from the GBD 2021 using the GBD results tool on the Institute for Health Metrics and Evaluation website. GBD 2021 provides estimates for 371 diseases and injuries across 204 countries and territories, 21 countries with subnational locations, for 25 age groups, over the years 1990–2021[23]. In GBD 2021, endometriosis case definitions were based on ICD-10 codes N80.0-N80.9 and corresponding ICD-9 codes 617-617.9. These codes comprehensively encompass uterine, ovarian, tubal, pelvic peritoneal, rectovaginal septum and vaginal, intestinal, cutaneous, as well as other specified or unspecified forms of endometriosis. We obtained incident cases, prevalent cases, and DALYS data for endometriosis from GBD 2021 databases. DALYs refers to the summation of years of life lost (YLLs) and years lived with disability (YLDs). YLLs are calculated as the estimated number of deaths and the product of the standardized life expectancy based on the age at death. YLDs are calculated by the prevalence of an individual consequence of a disease multiplied by its corresponding disability weight[24]. In addition, we also used sociodemographic index (SDI) to gauge social development in various countries and regions. The SDI is a composite index, defined as the geometric mean of three standardized indicators: the total fertility rate for women under age 25, mean years of schooling for those aged 15 years and older, and lag-distributed income per capita. This index reflects the level of social development at the national level across different countries and territories[25].

### Statistical analysis

We assessed the trends by age group using GBD 2021 age strata: 15–19, 20–24, 25–29, 30–34, 35–39, 40–44, 45–49, and 50–54 years. All rates were age-standardized using the direct standardization method by calling the age-adjust direct function from the epitools package. The standard population used for the standardization was the 2021 global standard population estimated by GBD^[23,26]^.

### Temporal trends

The joinpoint regression model identifies trends and key turning points in disease burden by calculating the sum of squared residuals between estimated and observed values. Compared to traditional linear trend analysis, joinpoint regression accurately captures dynamic changes in disease burden across different time periods[27]. We used joinpoint regression analysis to segment long-term trends into meaningful intervals, estimating the annual percentage change (APC), average annual percentage change (AAPC), and their 95% confidence intervals (CI). For ASIR, ASPR, and ASDR of endometriosis, an APC or AAPC > 0 with *P* < 0.05 indicated a significantly increasing trend, APC or AAPC < 0 with *P* < 0.05 indicated a decreasing trend, and a 95% CI including 0 with *P* ≥ 0.05 indicated a stable trend^[28,29]^. To comprehensively assess the long-term trends in endometriosis burden, we calculated the estimated annual percentage change (EAPC) as a key metric for evaluating temporal patterns. This approach was systematically applied to quantify trends in prevalence, incidence, and DALYs associated with endometriosis[30].

### Decomposition analysis

Decomposition analysis is a statistical methodology designed to quantify the relative contributions of diverse driving factors to temporal or spatial variations in endometriosis-related disease burden metrics, including incidence, prevalence and DALYs. Using the Das Gupta method, we decomposed changes in endometriosis burden from 1990 to 2021 into contributions from aging, population growth, and epidemiological changes. This approach provides a clearer understanding of how each factor has shaped the trends. Unlike traditional methods like linear regression, decomposition analysis isolates the independent effect of each factor on the overall burden change, helping identify key drivers of global trends^[31,32]^.

### Cross-country inequality analysis

Health inequality monitoring provides a foundation for evidence-based health planning and can inform policies, programs, and practices to reduce disparities in health distribution[33].The Slope Index of Inequality (SII) and the Concentration Index were used to quantify the burden inequality of endometriosis globally and across 21 GBD regions. The SII was calculated by regressing the age-standardized endometriosis rate on the SDI, using the midpoint of the cumulative population distribution ranked by SDI. To evaluate changes in health inequality, we analyzed data from 204 countries and territories between 1990 and 2021. A robust regression model (RLM), instead of an ordinary linear regression model, was applied to better manage bias and heterogeneity. This approach reduces sensitivity to outliers, minimizing bias from data variability or extreme values for a more accurate representation of health inequalities. Additionally, the concentration index was calculated by plotting the cumulative proportion of age-standard rate with the cumulative population distribution ranked by SDI and measuring the area under the Lorenz curve^[34,35]^.

### Bayesian Age-Period-Cohort model projection

Because the BAPC model can handle the complex, high-dimensional, and sparse data typical of large-scale epidemiological studies like GBD 2021, this approach was used to forecast the global burden of endometriosis from 2022 to 2040. The Integrated Nested Laplace Approximations (INLA) framework and the BAPC model were employed to predict future trends. The INLA framework was utilized alongside the BAPC model to approximate the marginal posterior distributions, effectively avoiding the mixing and convergence issues associated with conventional Bayesian methods that rely on Markov chain Monte Carlo sampling techniques^[36,37]^. These models assume the logit risk of incidence, prevalence and DALYS for a given age group and period can be modeled as a linear function of age, period, and cohort effects, thereby enabling an in-depth analysis of temporal changes in mortality. Furthermore, they allow for various combinations of age, period, and cohort influences, including constant or linear trends modeled as first- or second-order random walks, to capture diverse temporal dynamics^[38,39]^.The analyses is conducted using the default parameters in the BAPC package. The specific parameter information can be found at https://rdrr.io/rforge/BAPC/man/BAPC.html.

Perform Joinpoint regression by invoking the Joinpoint command-line version via function calls. The SII and Concentration Index were calculated through Stata 16.0. The preceding BAPC analysis is based on the R package “BAPC” (Version 0.0.36) and “INLA” (Version 23.09.09). Stata 16 and R software (Version 4.3.3) were used for data analysis and visualization and two-sided *P* < 0.05 was considered statistically significant.

The data analysis of this study was conducted from 1 November 2024 to 15 November 2024. AI was not used in the design, conduct, data analysis, or manuscript preparation of this study.

## Result

### Global level

**Figure 1.**
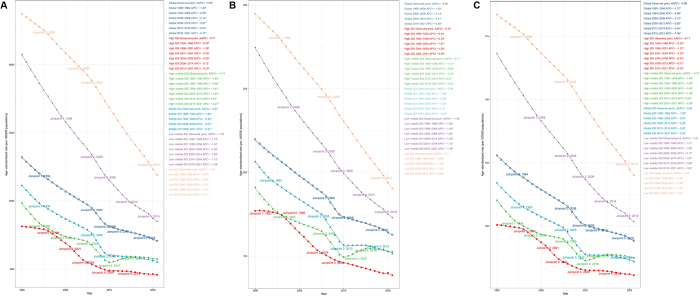
Temporal trend changes in ASPR (A), ASIR (B), and ASDR (C) for endometriosis globally and in various SDI regions from 1990 to 2021 based on the Joinpoint regression model.

In 2021, the global burden of endometriosis was substantial, with an estimated 22.28 million cases (95% UI: 13.67, 33.69), representing a 12.11% increase from 1990. Despite the rise in the absolute number, there was a slight decrease for the ASPR, from 1391.65 per 100 000 (95% UI: 842.39, 2129.67) in 1990 to 1023.8 per 100 000 (95% UI: 627.36, 1549.77) in 2021 (Table 1, Figure 1A). The global incidence in 2021 was 3.45 million cases (95% UI: 1.80, 5.64), marking a 3.51% increase from 1990. Correspondingly, the ASIR displayed a decline from 219.92 per 100 000 (95% UI: 113.45, 361.04) in 1990 to 162.71 per 100,000 (95% UI: 85.21, 265.35) in 2021. This downward trend in incidence is reflected by an EAPC for ASIR of −1 (95% CI: −1.05, −0.96) (Table 2, Figure 1B). The global DALYs due to endometriosis in 2021 totaled 2.05 million (95% UI: 1.11, 3.43), with an ASDR of 94.25 per 100 000 (95% UI: 50.82, 157.73) and an EAPC of −1.01 (95% CI: −1.06, −0.96) (Table S1 http://links.lww.com/JS9/E821, Figure 1C).

**Table 1:** The prevalence of endometriosis in 1990 and 2021, and changes from 1990 to 2021 at the global level and different regions

location	No. (95% UI) 1990	No. (95% UI) 2021	ASPR No. × 10^−5^ (95% UI)	ASPR No. × 10^−5^ (95% UI)	EAPC
Global	19 869 195.64 (11 990 157.75, 30 495 669.26)	22 275 015.21 (13 665 028.93, 33 685 040.5)	1391.65 (842.39, 2129.67)	1023.8 (627.36, 1549.77)	−1.02 (−1.07, −0.97)
High SDI	2 784 337.01 (1 678 168, 4 323 027.51)	2 495 271.47 (1 567 680.71, 3 697 457.56)	1087.6 (655.11, 1691.54)	874.89 (545.91, 1307.15)	−0.85 (−0.94, −0.76)
High-middle SDI	3 627 103.19 (2 172 525.53, 5 608 611.72)	3 410 962.02 (2 128 858.19, 5 094 600.79)	1190.63 (714.74, 1837.97)	944.37 (587.54, 1415.42)	−0.71 (−0.81, −0.62)
Middle SDI	5 892 115.28 (3 517 686.06, 9 102 898.61)	6 549 196.08 (4 002 699.48, 9 953 701.91)	1268.23 (760.61, 1951.64)	930.51 (567.48, 1416.85)	−1.04 (−1.12, −0.96)
Low-middle SDI	5 225 146.81 (3 156 078.22, 7 996 121.85)	6 091 634.17 (3 706 965.06, 9 312 503.86)	1845.32 (1118.04, 2808.57)	1109.54 (676.14, 1693.46)	−1.67 (−1.7, −1.64)
Low SDI	2 324 260.43 (1 403 825.98, 3 553 918.19)	3 710 059.28 (2 210 282.35, 5 680 442.28)	2022.88 (1224.21, 3077.64)	1314 (786.61, 2002.31)	−1.4 (−1.47, −1.32)
High-income Asia Pacific	699 270.28 (412 162.58, 1 078 919.64)	545 354.82 (340 224.94, 804 799.06)	1370.19 (805.94, 2120.76)	1174.55 (726.31, 1761.71)	−0.62 (−0.73, −0.5)
High-income North America	797 899.34 (449 704.4, 1 279 317.74)	594 499.2 (374 298.27, 874 670.98)	934.28 (525.39, 1502.48)	609.96 (381.66, 902.65)	−1.91 (−2.08, −1.75)
Western Europe	961 930.5 (574 435.53, 1 504 198.02)	900 964.47 (561 312.07, 1 371 058.82)	893.54 (531.89, 1403.72)	851.32 (526.04, 1310.62)	−0.08 (−0.1, −0.05)
Australasia	67 389.7 (40 323.84, 106 196.72)	80 842.14 (49 536.93, 124 254.89)	1138.39 (681.06, 1796.84)	991.62 (603.13, 1535.33)	−0.28 (−0.36, −0.2)
Andean Latin America	115 457.46 (69 203.37, 178 292.34)	155 218.84 (92 448.91, 242 314.08)	1172.61 (707.03, 1798.98)	801.11 (477.49, 1249.9)	−1.15 (−1.22, −1.07)
Tropical Latin America	472 933.7 (270 237.82, 760 208.8)	623 743.9 (383 238.37, 954 264.75)	1118.81 (642.14, 1791.11)	911.41 (559.57, 1396.39)	−1.06 (−1.29, −0.83)
Central Latin America	498 631.88 (300 192.12, 768 646.99)	575 740.85 (346 604.34, 888 888.32)	1146.12 (693.17, 1756.83)	760.38 (457.6, 1174.84)	−1.27 (−1.37, −1.18)
Southern Latin America	127 546.6 (74 874.98, 199 073.84)	159 083.96 (99 568.36, 233 997.67)	952.15 (559.97, 1482.56)	800.43 (499.17, 1181.74)	−0.41 (−0.53, −0.29)
Caribbean	110 052.33 (65 334.33, 171 653.91)	112 185.19 (66 316.06,176 035.4)	1109.12 (661.19, 1720.47)	832.44 (491.46, 1308.1)	−0.86 (−0.9, −0.83)
Central Europe	329 932.64 (204 292.97, 505 114.74)	260 161.75 (160 645, 397 475.63)	969.3 (600.28, 1484.2)	885.75 (546.61, 1358.24)	−0.22 (−0.36, −0.07)
Eastern Europe	1 038 437.89 (640 566.25, 1 577 704.6)	882 964.32 (544 948.56, 1 342 805.72)	1618.68 (997.4, 2460.79)	1585.38 (977.34, 2415.93)	0.32 (0.15, 0.5)
Central Asia	238 771.42 (146 243.83, 367 485.14)	278 735.7 (172 217.95, 424 801.53)	1285.56 (787.62, 1973.81)	1017.38 (627.87, 1550.01)	−0.43 (−0.62, −0.25)
North Africa and Middle East	1 504 316.73 (899 207.97, 2 308 291.14)	2 200 421.08 (1 326 806.04, 3 377 916.73)	1847.19 (1112.88, 2815.54)	1258.23 (759.35, 1930.54)	−1.3 (−1.36, −1.24)
South Asia	4 929 562.83 (2 990 875.79, 7 490 020.67)	5 866 273.34 (3 561 973.73, 8 968 531.5)	1849.61 (1124.62, 2796.92)	1087.35 (661.04, 1660.22)	−1.77 (−1.8, −1.74)
Southeast Asia	1 932 516.3 (1 192 520.09, 2 939 459.06)	2 420 576.08 (1 479 728.44, 3 662 611.18)	1533.41 (948.98, 2322.65)	1176.58 (718.53, 1782.27)	−0.8 (−0.83, −0.77)
East Asia	4 003 165.85 (2 282 924.05, 6 303 923.54)	3 116 327.55 (1 901 911.29, 4 693 324.04)	1158.72 (666.05, 1816.93)	752.81 (454.4, 1142.13)	−1.53 (−1.7, −1.35)
Oceania	35 141.66 (20 938.1, 54 210.72)	67 931.47 (39 387.58, 103 825.07)	2209.61 (1325.2, 3381.62)	1832.2 (1066.77, 2790.6)	−0.59 (−0.61, −0.57)
Western Sub-Saharan Africa	828 383.98 (505 017.68, 1 270 317.38)	1 620 504.96 (982 404.45, 2 439 381.17)	1853.09 (1134.14, 2823.16)	1313.22 (798.96, 1969.77)	−1.05 (−1.11, −1)
Eastern Sub-Saharan Africa	738 162.75 (446 995.22, 1 138 694.89)	1 148 923.38 (681 259.58, 1 768 902.18)	1679.14 (1020.55, 2575.18)	1049.89 (626.71, 1606.38)	−1.51 (−1.56, −1.45)
Central Sub-Saharan Africa	246 937.37 (147 743.33, 381 071.67)	405 555.44 (240 277.49, 633 287.46)	1932.61 (1158.93, 2961.15)	1204.54 (717.92, 1873.09)	−1.44 (−1.57, −1.3)
Southern Sub-Saharan Africa	192 754.42 (117 147.03, 295 505.13)	259 006.76 (156 887.64, 396 097.81)	1390.61 (847.43, 2121.95)	1084.29 (656.94, 1656.06)	−0.75 (−0.79, −0.72)

ASPR, age-standardized prevalence rates; EAPC, estimated annual percentage change; SDI, Socio-demographic Index.

**Table 2:** The incidence of endometriosis in 1990 and 2021, and changes from 1990 to 2021 at the global level and different regions

location	No. (95% UI) 1990	No. (95% UI) 2021	ASR No. × 10^−5^ (95% UI) 1990	ASR No. × 10^−5^ (95% UI) 2021	EAPC
Global	3 330 200.05 (1 737 295.21, 5 432 158.31)	3 447 125.88 (1 795 822.76, 5 636 732.96)	219.92 (113.45, 361.04)	162.71 (85.21, 265.35)	−1 (−1.05, −0.96)
High SDI	437 908.4 (216 854.7, 742 189.45)	358 019.31 (180 686.32, 605 212.39)	176.99 (88.08,299.58)	138.51 (70.91, 233.6)	−0.92 (−0.99, −0.85)
High-middle SDI	593 606.46 (302 244.77, 979 980.51)	488 882.81 (248 635.12, 806 322.97)	191 (96.75, 316.37)	152.69 (79.85, 248.32)	−0.72 (−0.82,-0.62)
Middle SDI	1 049 820.76 (542 005.09, 1 713 276.38)	1 008 051.89 (516 152.71, 1 657 623.21)	206.38 (104.83, 339.96)	151.46 (78.47, 247.69)	−1.04 (−1.12, −0.97)
Low-middle SDI	863 042.96 (458 621.65, 1 396 094.88)	981 567.48 (512 392.16, 1 598 015.85)	274.73 (143.12, 449.11)	173.02 (89.73, 282.72)	−1.5 (−1.52, −1.47)
Low SDI	383 153.46 (204 645.54, 618 792.09)	607 883.49 (321 234.42, 993 013.98)	294.71 (153.79, 482.12)	190.36 (98.35, 314.8)	−1.41 (−1.46, −1.36)
High-income Asia Pacific	110 470.72 (53 649.76, 185 277.98)	77 235.23 (37 586.51, 131 144.43)	221.02 (107.33, 370.06)	191.9 (94.86, 325.39)	−0.53 (−0.63, −0.42)
High-income North America	133 506.05 (64 090.74, 232 842.97)	91 185.88 (45 320.03, 153 289.74)	159.6 (76.58, 279.24)	99.5 (49.71,167.14)	−2.07 (−2.24, −1.9)
Western Europe	140 947.05 (66 717.49, 251 893.26)	126 349.52 (60 855.51, 223 814.55)	137.35 (65.52, 245.78)	133.17 (65.18, 235.44)	0.02 (−0.02, 0.07)
Australasia	10 749.31 (5188.26, 18 598.09)	12 016.69 (5663.16,21 195.61)	185.13 (89.59, 320.48)	162.48 (77.59, 286.43)	−0.25 (−0.33, −0.18)
Andean Latin America	20 893.91 (10 723.34, 34 288.3)	25 037.61 (12 522.52, 41 937.44)	191.73 (96.94, 317.28)	130.34 (65.34, 218.29)	−1.19 (−1.26, −1.12)
Tropical Latin America	82 433.87 (41 918.13, 137 283.36)	90 680.83 (45 659.38,150 050.8)	182.85 (92.06, 306.62)	139.67 (71.35, 229.43)	−1.2 (−1.36, −1.04)
Central Latin America	88 215.24 (46 299.54, 144 072.71)	89 764.2 (44 966.84, 150 400.19)	181.98 (93.64, 300.12)	120.05 (60.32, 200.84)	−1.3 (−1.39, −1.21)
Southern Latin America	21 125.4 (10 158.08, 36 669.69)	24 586.6 (12 235.02, 41 415.95)	154.66 (74.21, 268.28)	128.51 (64.26, 216.54)	−0.47 (−0.56, −0.38)
Caribbean	18 434.87 (9322.78, 30 460.59)	17 085.68 (8494.58, 28 773.17)	173.31 (86.57, 288.27)	130.23 (65.04, 218.74)	−0.88 (−0.91, −0.86)
Central Europe	50 948.64 (26 719.49, 83 359.62)	36 129 (18 469.42, 60 454.17)	155.28 (82.35, 252.68)	140.23 (74.2, 230.17)	−0.23 (−0.37, −0.09)
Eastern Europe	148 323.91 (77 945.08, 242 575.48)	117 894.39 (60 182.16, 195 821.6)	250.77 (133.85, 407.59)	245.6 (130.95, 400.14)	0.32 (0.15,0.5)
Central Asia	39 426.46 (21 388.3, 63 492.51)	42 300.27 (22 244.37, 68 867.24)	204.22 (108.93, 331.43)	163.65 (87.02, 265.19)	−0.39 (−0.57, −0.21)
North Africa and Middle East	259 963.65 (135 394.49, 421 780.61)	341 353.71 (177 477.33, 560 634.57)	283.25 (144.85, 463.72)	196.04 (102.21, 321.78)	−1.19 (−1.29, −1.09)
South Asia	801 050.14 (432 071.79, 1 299 042.31)	936 793.56 (491 400.06, 1 530 317.1)	274.03 (145.06, 448.39)	169.55 (88.49, 277.83)	−1.57 (−1.6, −1.55)
Southeast Asia	341 472.08 (182 807.84, 550 563.79)	386 144.63 (199 088.06, 628 758.16)	250.09 (131.68, 406.46)	192.96 (99.97, 313.41)	−0.79 (−0.82, −0.76)
East Asia	704 502.43 (347 104.09, 1 187 985.57)	444 699.34 (216 496.14, 747 183.83)	189.07 (92.11, 321.13)	123.14 (61.32, 204.64)	−1.51 (−1.68, −1.34)
Oceania	5878.65 (3027.84, 9571.11)	10 521.9 (5149.62, 17 539.73)	333.69 (168.36, 549.05)	272.59 (132.38, 455.76)	−0.63 (−0.65, −0.61)
Western Sub-Saharan Africa	146 209.07 (78 638.01, 234 660.06)	272 483.35 (145 767.84, 439 859.43)	284.72 (149.5, 463.25)	193.71 (101.29, 317.05)	−1.22 (−1.28, −1.16)
Eastern Sub-Saharan Africa	130 255.89 (69 663.72, 210 496.14)	195 550.44 (101 129.3, 319 181.83)	256.65 (133.85, 420.64)	156.54 (79.18,258.89)	−1.59 (−1.64, −1.54)
Central Sub-Saharan Africa	41 891.54 (22 349.79, 67 582.28)	68 807.4 (35 581.77, 113 066.85)	289.34 (150.86, 473.45)	182.02 (92.32, 303.02)	−1.42 (−1.54, −1.31)
Southern Sub-Saharan Africa	33 501.18 (17 953.43, 53 949.5)	40 505.65 (20 951.67, 66 730.86)	215.48 (113.05, 351.33)	169.3 (87.58, 279.34)	−0.75 (−0.77, −0.72)

ASIR, age-standardized incidence rates; EAPC, estimated annual percentage change; SDI, Sociodemographic Index.

### Regional level

There were significant regional differences in endometriosis burden, strongly correlated with SDI levels. Low SDI regions experienced the highest ASPR at 1314 per 100 000 (95% UI: 786.61, 2002.31), whereas high SDI regions displayed the lowest ASPR at 874.89 per 100 000 (95% UI: 545.91, 1307.15) (Table 1 and Figure S1A http://links.lww.com/JS9/E821, Figure 1A). In these regions, the ASPR decreased from 2022.88 per 100 000 (95% UI: 1224.21, 3077.64) in 1990 to 1314 per 100 000 (95% UI: 786.61, 2002.31) in 2021. Low-middle SDI regions exhibited the greatest decline in ASPR, with an EAPC of −1.67 (95% CI: −1.7, −1.64) (Table 1 and Figure S1A http://links.lww.com/JS9/E821, Figure 1A).

Regional patterns for ASIR and ASDR were similar. Low SDI regions reported the highest ASIR and ASDR, while high SDI regions had the lowest rate. For example, the ASIR in low SDI regions was 190.36 per 100 000 (95% UI: 98.35, 314.8), compared to 138.51 per 100 000 (95% UI: 70.91, 233.6) in high SDI regions (Table 2 and Figure S1B http://links.lww.com/JS9/E821, Figure 1B). Likewise, the ASDR in low SDI regions was 120.08 per 100,000 (95% UI: 62.99, 200.07), versus 80.58 per 100,000 (95% UI: 43.37, 134.5) in high SDI regions (Table S1 http://links.lww.com/JS9/E821 and Figure S1C http://links.lww.com/JS9/E821, Figure 1C). Together, these findings highlighted the complex relationship between sociodemographic status and endometriosis burden. While high SDI regions have made significant progress in reducing the prevalence and impact of endometriosis, low SDI regions still face significant challenges.

ASPR declined in most regions but increased in Eastern Europe, with an EAPC of 0.32 (95% CI: 0.15, 0.5). High-income North America experienced the largest decline in ASPR, reaching 609.96 per 100 000 (95% UI: 381.66, 902.65) in 2021, and the EAPC was −1.91 (95% CI: −2.08, −1.75), followed by South Asia with an EAPC of −1.77 (95% CI: −1.8, −1.74). Oceania demonstrated the highest ASPR, with 1832.2 per 100 000 (95% UI: 1066.77, 2790.6), followed by Eastern Europe, with 1585.38 per 100 000 (95% UI: 977.34, 2415.93) (Table 1, Figure S1A http://links.lww.com/JS9/E821). Oceania and Eastern Europe also had the highest ASIR. Specifically, Oceania reported an ASIR of 272.59 per 100 000 (95% UI: 132.38, 455.76), while Eastern Europe had an ASIR of 245.6 per 100 000 (95% UI: 130.95, 400.14) . In contrast, High-income North America exhibited the lowest ASIR at 99.5 per 100 000 (95% UI: 49.71, 167.14) and highest decline with EAPC of −2.07 (95% CI: −2.24, −1.9). Only Eastern Europe and Western Europe experienced an increase in ASIR, with an EAPC of 0.32 (95% CI: 0.15, 0.5) and 0.02 (95% CI: −0.02, 0.07), respectively (Table 2, Figure S1B http://links.lww.com/JS9/E821). From 1990 to 2021, the ASDR decreased the most in high-income North America (EAPC: −1.92, 95% CI: − 2.08, −1.76) and the least in Western Europe (EAPC −0.08, 95% CI: −0.1, −0.05). Eastern Europe was the only region with an increasing ASDR, with an EAPC of 0.33 (95% CI: 0.15, 0.5). In 2021, Oceania and Eastern Europe had the highest ASDR at 168.52 (95% UI: 84.58, 290.53) and 146.28 (95% UI: 78.19, 245.1) per 100 000 (Table S1 http://links.lww.com/JS9/E821, Figure S1C http://links.lww.com/JS9/E821).

### Country level

The ASPR of endometriosis among 204 countries varied from 518.54 to 2383.43 per 100 000. Niger (2383.43 per 100 000; 95% UI: 1393.25, 3600.93), Chad (1992.29 per 100 000; 95% UI: 1155.19, 3030.03), and Solomon Islands (1985.24 per 100 000; 95% UI: 1153.28, 3015.55) exhibited the highest ASPR. Notably, two of these countries are in Africa (Niger and Chad), and one is in Oceania (Solomon Islands). The largest decrease in ASPR from 1990 to 2021 was observed in Yemen. Seventeen countries experienced an increase in ASPR, among which, Iceland stood out with the highest EAPC of 1.21 (95% CI: 0.93, 1.48) (Table S2 http://links.lww.com/JS9/E821). Solomon Islands had the highest ASIR (285.04 per 100 000; 95% UI: 139.87, 475.66), while Iceland had the lowest rate (79.09 per 100 000; 95% UI: 36.84, 147.04). The wide range of incidence rate indicates that risk factors, healthcare systems, and prevention strategies vary between countries. Seventeen countries experienced an increase in ASIR, again, Iceland was the most prominent, with an EAPC of 2.4 (95% CI: 2.02, 2.78) (Table S2 http://links.lww.com/JS9/E821). In 2021, India had the highest DALYs due to endometriosis, with 392 961.05 (95% UI: 208 162.52, 661 651.09), and Niger displayed the highest ASDR at 218.81 per 100 000 (95% UI: 109.26, 366.38). Solomon Islands ranked second in ASDR (182.53 per 100 000; 95% UI: 91.42, 315.46), and China ranked second in DALYs with 269 427.74 (95% UI: 142 542.44, 455 047.13). The ASDR decreased the most from 1990 to 2021 in Yemen (EAPC −2.83, 95% CI: −3.02, −2.63), Nepal (EAPC −2.62, 95% CI: −2.73, 2.5.), and Equatorial Guinea (EAPC −2.56, 95% CI: −2.64, −2.48). Seventeen countries experienced an increase in ASDR, Iceland again had the largest rise, with an EAPC of 1.21 (95% CI: 0.93, 1.48) (Table S2 http://links.lww.com/JS9/E821). The consistency between national and regional patterns highlightes the significant challenges posed by endometriosis in parts of Oceania and Africa, impacting both prevalence and overall health-related quality of life in those populations.

### Age patterns

**Figure 2.**
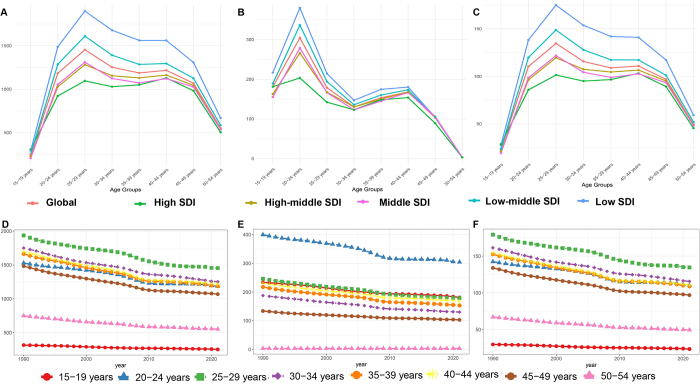
The rate of prevalence (A), incidence (B), and DALYs (C) of endometriosis in 1990 and 2021 at different age, and the rate of prevalence (D), incidence (E), and DALYs (F) of endometriosis over time by age category..

The age distribution of endometriosis metrics (prevalence, incidence, and DALYs) was largely consistent across different SDI regions. Both prevalence and DALYs rates peaked at 25–29 year age group, with a slight secondary increase at 40–44 years. By contrast, the incidence was highest in the 20–24 year age group, then declined through ages 30–34 years, followed by a second small peak at 40–44 years, and finally a significant decline after 45 years (Table S3 http://links.lww.com/JS9/E821). These patterns suggest that the greatest burdens of endometriosis falls in women aged 25–29 years. From 1990 to 2021, prevalence, incidence and DALYs rates declined across all age groups. Throughout this period, women aged 25–29 years maintained the highest prevalence and DALY rates, while women aged 20–24 years consistently had the highest incidence rates (Figure 2D-F, Table S4-6 http://links.lww.com/JS9/E821).

### Decomposition analysis

**Figure 3.**
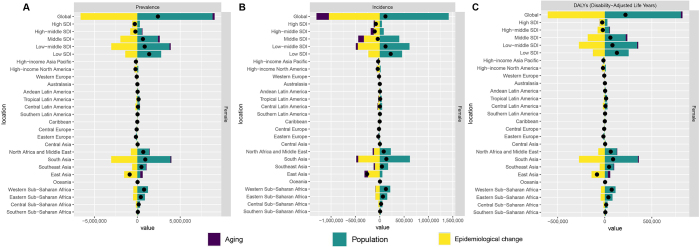
Change in prevalence (A), incidence (B), and DALYs (C) of endometriosis decomposed by three population-level determinants: aging, population and epidemiological change at the global level, five regions, and and 21 GBD regions.

The incidence, prevalence and DALYs of endometriosis attributable to aging, population and epidemiologic change were analyzed by decomposition analysis at the global level. Subgroup analyses were performed across five SDI strata and 21 regions, encompassing a total of 27 individual areas. Globally, population growth accounted for a 364.86% change for prevalence, followed by epidemiologic change (−274.67%) and aging (9.81%). Similarly, population growth alone would account for 1211.68% of the incidence change and 366.99% of the DALYs change (Figure 3, Table S7 http://links.lww.com/JS9/E821). In low SDI regions, population growth was an overwhelming driver of prevalence change (200.93%), whereas epidemiological change contributed −100.89% and aging contributed −0.03%. In low-middle SDI regions, population growth was the largest contributor to changes in prevalence and DALYs, while in high and high-middle SDI regions, most of the change was due to epidemiological improvements. Population and epidemiological change also substantially influenced changes in prevalence, incidence, and DALYs in South Asia (Figure 3, Table S7 http://links.lww.com/JS9/E821).

### Cross-country health inequality analysis

**Figure 4.**
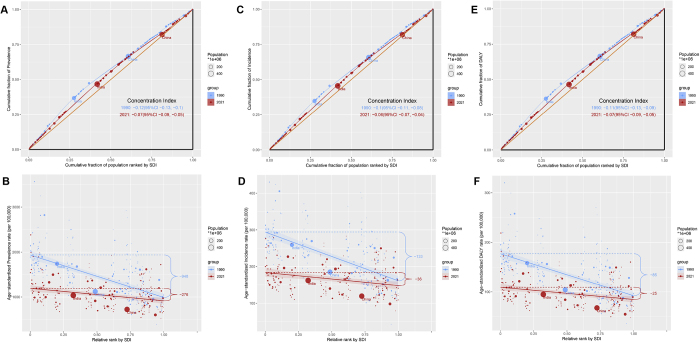
Health inequality regression curves and concentration curves for the prevalence (A–B), incidence (C–D), and DALYs (E–F) of endometriosis.

These findings reveal significant inequalities in endometriosis burden between higher- and lower-SDI regions, in both absolute and relative terms. The concentration index of prevalence improved from −0.12 (95% CI: −0.13, −0.1) in 1990 to −0.07 (95% CI: −0.09, −0.05) in 2021 (Figure 4A). Consistently, the SII for prevalence between high and low SDI regions narrowed from −948 in 1990 to −276 in 2021 (Figure 4B). Similarly, the concentration index for incidence improved from −0.1 (95% CI: −0.11, −0.08) in 1990 to −0.06 (95% CI: −0.07, −0.04) in 2021 (Figure 4C), and the SII improved from −133 to −36 (Figure 4D). For DALYs, the concentration index went from −0.11 (95% CI: −0.13, −0.09) to −0.07 (95% CI: −0.09, −0.05) (Figure 4E), and the SII of DALYs improved from −85 to −25 (Figure 4F). These results demonstrate a reduction in the inequality of endometriosis burden over time.

### Trend of endometriosis from 2022 to 2040

**Figure 5.**
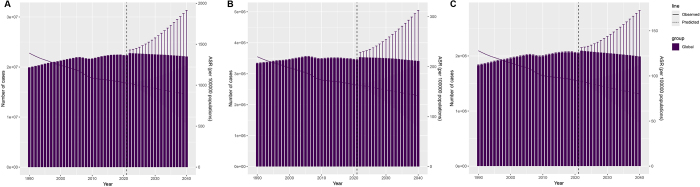
Trend in ASPR (A), ASIR (B), and ASDR (C) of endometriosis from 2022 to 2040. The purple line represents the predicted trend, and the gray-shaded area represents the 95% CI of the predicted values; the gray-dotted vertical line divides the data into real values (1990–2021) and predicted values (2022–2040).

The burden of endometriosis is projected to change significantly from 2021 to 2040. The global ASPR is also projected to decline, from 1023.8 per 100 000 (95% CI: 1023.38, 1024.23) in 2021 to 887.89 per 100 000 (95% CI: 530.79, 1245) by 2040 (Figure 5A, Table S8 http://links.lww.com/JS9/E821)Th.e ASIR is expected to decrease globally from 162.71 per 100 000 (95% CI: 162.54, 162.88) in 2021 to 142.13 per 100 000 (95% CI: 77.93, 206.33) by 2040, representing a 12.65% decrease (Figure 5B, Table S8 http://links.lww.com/JS9/E821). Finally, the ASDR is forecast to decrease from 94.25 per 100 000 (95% CI: 94.12, 94.38) in 2021 to 80.03 per 100 000 (95% CI: 47.59, 112.47) by 2040 (Figure 5C, Table S8 http://links.lww.com/JS9/E821).

## Discussion

Our comprehensive analysis of endometriosis burden from 1990 to 2021 reveals a complex pattern of disparities and trends across regions and SDI levels. Globally, disease burden of endometriosis declined over this period. The burden varied across income levels and was inversely related to income level. Correspondingly, health inequalities among the relevant population have improved. Population growth and epidemiological change were identified as the two major drivers of changes in prevalence, incidence and DALYs. Notably, women aged 25–29 years emerged as a particularly affected group, underscoring the necessity for early screening and diagnose, as well as early laparoscopic interventions that preserve fertility and protect nerve function in this age group. Overall, this study highlights the global health impact of endometriosis and provides a critical foundation for developing prevention and management strategies, such as targeted surgical resource planning, tiered intervention strategies in low-resource settings, and the development of cost-effective prevention and management methods.

Our observed decreasing trends are consistent with previous GBD studies, which also reported declines in age-standardized rates of endometriosis over time^[18,20]^, reflecting significant advances in women’s health in recent decades. One example of progress is the the National Action Plan for Endometriosis (NAPE), launched in 2018 to improve patients’ outcomes via increased awareness and education, better clinical management, and research[40]. Similarly, the 2021 WHO Endometriosis Fact Sheet was a welcome step toward raising the global awareness of the disease. The success of NAPE, along with other global efforts, has contributed to significant reductions in the prevalence and incidence rate, especially in low-income regions with a heavy endometriosis burden[41]. Nevertheless, high SDI regions still show the lowest ASPR, ASIR and ASDR while low SDI regions have the highest rates, indicating the persistent socioeconomic disparities in the global burden of endometriosis. This disparity likely results from a combination of factors, including healthcare access, public health systems, and geographical, cultural, and socioeconomic differences, as well as environmental and genetic issues[3]. The diagnosis of endometriosis typically relies on imaging (e.g., transvaginal ultrasound and magnetic resonance imaging) or laparoscopic examination[42], which are often scarce or unevenly distributed in low SDI regions. Additionally, the lack of specialized doctors and surgical equipment in low SDI areas makes it difficult for patients to access effective treatment, thereby increasing the burden, especially the DALYs[43]. The etiologies of endometriosis are complex, for example, prenatal exposure to diethylstilbestrol[44], low birthweight[45], lean childhood body size[46], low body mass index, and waist-to-hip ratio^[46,47]^ have all been associated with a higher incidence of endometriosis. These risk factors are more common in low SDI populations. Women in low SDI regions may face more severe environmental pollution issues, and environmental pollutants such as dioxins and polychlorinated biphenyls are considered potential risk factors for endometriosis[48]. In low SDI regions, poor dietary habits (such as high-fat, low-fiber diets) and malnutrition[49], coupled with a higher likelihood of prolonged exposure to pesticides, chemicals, or other harmful substances[50], thereby increasing the risk of developing the disease. Certain genetic variants may also elevate the risk of endometriosis[51], and the distribution of these genetic variants may vary among different populations. Additionally, the impact of genetic factors may be mitigated by better medical management and environmental conditions[52]. Indeed, the prevalence rates in low SDI regions may even be underestimated, socioeconomic barriers, lack of awareness, insufficient medical resources, and inadequate provider training all contribute to diagnostic delays and missed diagnoses^[53,54]^. The persistently high burden in low SDI regions warrants further consideration. Implementing measures such as primary healthcare training programs, public health education campaigns, and accessible non-invasive diagnostic facilities may substantially mitigate these issue. In contrast, high SDI regions benefit from stronger healthcare systems and effective public health interventions, which likely reduce the endometriosis burden. The exceptionally high prevalence and incidence rates observed in Oceania and Eastern Europe might due to a combination of socioeconomic factors, ethnicity, and education level differences^[55-57]^. These regions urgently need targeted public health policies to raise awareness, implement multidisciplinary care (such as postoperative follow-up, psychological and social support), and invest in research into etiology, biomarker discovery, and effective treatment.

Laparoscopic surgery holds an irreplaceable and critical position in the diagnostic and therapeutic system for endometriosis. As the clinical diagnostic gold standard, laparoscopic technology enables precise visual assessment of pelvic lesions, comprehensively reflecting the anatomical distribution and severity of the disease[58]. In terms of treatment, laparoscopic surgery effectively alleviates pain symptoms and improves reproductive outcomes through accurate excision or ablation of ectopic lesions[59]. Its unique minimally invasive advantages facilitate meticulous dissection of pelvic adhesions and restoration of normal anatomical relationships, thereby significantly reducing the risk of disease recurrence. However, the clinical efficacy of this technique is strongly correlated with the surgeon’s experience[60] and exhibits notable regional disparities. High and middle SDI regions demonstrate higher adoption rates of laparoscopic surgery, whereas low SDI regions face diagnostic and therapeutic challenges[61], primarily due to limited healthcare resource allocation and technical accessibility barriers[62]. Notably, there is no unified standard for surgical approaches (e.g., extent of resection, application of specific techniques) across regions with varying development levels. High SDI regions have progressively incorporated ultrasound-based preoperative staging systems to assess surgical complexity[63] and adopted advanced technologies such as robot-assisted laparoscopy. In contrast, middle and low SDI regions still rely predominantly on conventional laparoscopic techniques[64].This regional disparity underscores the necessity for stratified diagnostic and therapeutic strategies. In low SDI regions, a three-tier intervention strategy is recommended. First, establish resource allocation optimization mechanisms; second, improve community hospital-based early screening systems; and finally, implement comprehensive measures including establishing specialized treatment centers, developing tiered physician training programs, and creating digital remote mentoring platforms to systematically enhance laparoscopic surgical skills for endometriosis. International collaborations (e.g., initiatives by the International Federation of Gynecology and Obstetrics) can provide support through donated second-hand equipment and training programs. Additionally, promoting cost-effective techniques such as traditional laparoscopic electrocoagulation combined with blunt dissection, reducing reliance on high-cost consumables[65], may serve as a feasible solution for resource-limited settings. Given the burden in women aged 25–29, surgeons should prioritize fertility-preserving and nerve-sparing techniques, actively promote the widespread adoption of these procedures, and explore novel surgical approaches[66].

To elucidate the underlying factors contributing to the evolving disease burden of endometriosis from 1990 to 2021, the study conducted decomposition analysis based aging, population growth and epidemiological changes. The results revealed that population expansion and epidemiologic change significantly influenced the variations in prevalence, incidence and DALYs of endometriosis across different SDI region. Specifically, the global population growth has been identified as a primary driver of increased disease burden. Conversely, the epidemiological change have demonstrated a mitigating effect on the global burden of endometriosis, as evidenced by reductions in prevalence, incidence and DALYs. This positive trend suggests that public health initiatives and medical interventions are yielding measurable progress. However, it is noteworthy that while epidemiological changes have reduced the burden both globally and within all five SDI regions, these improvements have been substantially counterbalanced by population growth. To effectively manage and control the disease burden of endometriosis, it is imperative to develop strategies that optimize the allocation and utilization of public health resources, particularly in the context of ongoing population growth.

Few studies have investigated the incidence and prevalence of endometriosis in adolescents. One study found that among adolescents with pelvic pain, the prevalence of visually confirmed endometriosis (via laparoscopy) ranged from 25% to 100%. On average, about 49% of adolescents with chronic pelvic pain and 75% of those with pain unresponsive to medication were diagnosed with endometriosis[67]. Similarly, nearly two-thirds of adolescents with chronic non-cyclic pelvic pain were found to have endometriosis upon laparoscopy[67]. In our data, the peak prevalence and DALYs rates of endometriosis occurred in 25–29 year age group, whereas the highest incidence rate was in the 20–24 year group,. This disparity might be explained by an average diagnostic delays of 3–10 years for endometriosis[2]. One major reason for diagnostic delay is that laparoscopic surgery with biopsy has long been the gold standard, and its accuracy depends greatly on the surgeon’s skill and interest in detecting endometriosis^[42,68]^. In addition, many women hesitate to report symptoms due to embarrassment or the mistaken belief that their pain is “normal,” which further delays seeking medical attention^[13,69]^. Encouragingly, non-invasive diagnosis based on imaging technologies has improved the detection rate of endometriosis^[70,71]^, which is now recommended by clinical guidances[72]. Reportedly, the median age at symptom onset for endometriosis is 25 years, and the median age at diagnosis is 26 years[73], which is broadly consistent with our findings. However, other studies have reported different results. For example, the peak age at first diagnosis or suspicion of endometriosis was 30–34 years in Australian[74]. The highest prevalence rate was reported in the 35–44 year age group in Spain[75] and in the 36–45 year group in the USA[76]. Additionally, one study reported that the incidence rates declined over time in all age groups except those aged 26–30 and 56–60 years[76], a pattern inconsistent with our findings. Collectively, these observations reflect regional differences in the epidemiology of endometriosis, and reinforce that women aged 25–29 years merit particular attention in monitoring and management efforts. Patients with endometriosis frequently suffer from psychological conditions such as anxiety and depression[8], particularly women aged 25-29, who face significant fertility pressures and societal expectations, exacerbating their mental health challenges. The chronic pelvic pain associated with endometriosis severely impair women’s work capacity and productivity[77]. Given that women in this age group are at a pivotal stage of career development, the disease can have long-term consequences on their professional trajectories. Moreover, endometriosis is a leading cause of infertility[5], and women aged 25–29 years are in their prime reproductive years. The condition’s impact on their fertility may further contribute to declining birth rates. Therefore, it is imperative for the government to enhance policy advocacy to raise awareness of endometriosis among both the public and healthcare providers. Targeted preoperative screening (e.g., symptom checklists and imaging), standardized pain and fertility questionnaires should be incorporated into pre-pregnancy healthcare services for women in the most affected age groups to facilitate timely detection and treatment of endometriosis. Additionally, reproductive health education and counseling should be promoted to help patients better understand the disease’s implications for fertility and the available management options.

Despite the downward trend in endometriosis incidence and projections of further decline by 2040, the overall disease burden of endometriosis remains very high. Therefore, greater emphasis should be placed on the prevention, monitoring, diagnosis and treatment of endometriosis. As endometriosis is progressive, early diagnosis and treatment are particularly important for younger patients^[78,79]^. Endometriosis is also a leading cause of disability and reduced quality of life in women of reproductive age[43], underlining the need for timely intervention. In addition, delayed diagnosis can lead to central sensitization, chronic pain, and scarring^[43,80]^. Unfortunately, a lack of awareness about menstrual health and endometriosis among healthcare providers remains widespread. Educating both medical professionals and patients and their caregivers about what constitutes abnormal pelvic pain in adolescence is indispensable for promoting appropriate care-seeking behavior and reducing diagnostic delays in endometriosis^[43,81,82]^.

Our study has several advantages. First, to our knowledge, it is the first to analyze endometriosis burden by using cross-country inequality and decomposition analysis, which helped identify areas require greater attention and resources, and can guide optimal care allocation globally. Second, we utilized the newly released GBD 2021 dataset, which has a robust design, a large sample size, and sophisticated statistical methodology. Third, for the first time, we performed a detailed age-subgroup analysis of endometriosis burden for the first time, providing policymakers insight into which age groups to focus on.

However, there are still several limitations. First, the estimates of endometriosis in the GBD data may be influenced by multiple factors: the lack of unified diagnostic criteria globally, regional variations in disease coding systems, and inadequate medical surveillance systems, particularly in resource-limited settings, all of which contribute to case underestimation and reporting bias. Second, clinical diagnosis is challenging due to its diverse and atypical symptomatology, making traditional diagnostic approaches based on medical history and physical examination prone to misdiagnosis or missed diagnosis. Third, since GBD 2021 dose not provide data on the risk factors of endometriosis, so we could not assess the impact of relevant risk factors or adjust for potential confounding variables such as healthcare accessibility and socioeconomic status in our analysis. It is important to note that the GBD modeling methodology has inherent limitations. The accuracy of its estimates heavily depends on the availability and quality of primary data, yet there are substantial disparities in basic healthcare data collection capabilities across countries, particularly affecting low- and middle-income nations. Specifically, in regions with underdeveloped healthcare infrastructure and insufficient policy frameworks, the true incidence rates of endometriosis are likely to be substantially underestimated. Conversely, in some countries, the epidemiological data may present an overestimated disease burden, primarily due to the disproportionate reliance on data collected from major urban centers, which may not accurately represent the national disease profile[83]. Such data disparities may affect the accuracy and generalizability of our finding. Furthermore, our predictive model is based on historical APC trends and assumes constant healthcare conditions, diagnostic criteria, and public health policies. However, in reality, rapid advancements in diagnostic and surgical technologies, as well as evolving public health policies, may cause actual epidemiological patterns to deviate from current projections. These factors must be carefully considered when interpreting the results.

## Conclusion

In summary, our study shows that although ASPR, ASIR, and ASDR for endometriosis declined from 1990 to 2021, significant inequalities persist at global and regional levels. Countries with lower SDI experience a greater burden of endometriosis. In these countries, timely and effective prevention and management strategies are needed to improve global health outcomes. Internationally, it is critical to promote economic development in low-SDI countries, and for high-income regions to share advanced medical technologies and expertise to improve health outcomes. Additionally, more attention should be paid to the monitoring and management of endometriosis in women aged 25–29 years.

## Data Availability

The datasets analyzed are available in the GBD 2021.
